# An education intervention delivered to nursing faculty: an acceptability and feasibility study

**DOI:** 10.1590/0034-7167-2025-0218

**Published:** 2026-08-03

**Authors:** Rafaela Batista dos Santos Pedrosa, Andressa Teoli Nunciaroni, Suzanne Fredericks

**Affiliations:** I Universidade Estadual de Campinas. Campinas, São Paulo, Brazil; II Universidade Federal do Estado do Rio de Janeiro. Rio de Janeiro, Rio de Janeiro, Brazil; III Toronto Metropolitan University. Toronto, Ontario, Canada

**Keywords:** Education, Feasibility Studies, Knowledge, Nursing, Nursing Faculty., Educação, Estudos de Viabilidade, Conhecimento, Enfermagem, Corpo Docente de Enfermagem., Educación, Estudios de Factibilidad, Conocimiento, Enfermería, Docentes de Enfermería.

## Abstract

**Objectives:**

to determine the acceptability and feasibility of a health-related program planning educational intervention delivered to nursing faculty in Brazil, comparing preand post-learning knowledge.

**Methods:**

a feasibility study conducted at two Brazilian universities. Sixteen professors were recruited and underwent a multi-component intervention that addressed the content in two phases: ten pre-recorded sessions and readings; five live interactive online sessions. Knowledge and acceptability data were analyzed descriptively, and t-test analyzes were also calculated to determine the difference in knowledge and ability scores.

**Results:**

the findings indicate that participants considered the educational intervention both acceptable and feasible. However, they also expressed concerns regarding insufficient time to fully engage with the intervention due to work-related responsibilities, emphasizing the need for an extra question-and-answer session to enhance support for content practical application.

**Conclusions:**

our findings suggest the intervention is acceptable and feasible, and will support expanding assessment tests through a large-scale clinical trial.

## RESUMO

**Objetivos:**

determinar a aceitabilidade e viabilidade de uma intervenção educacional em planejamento de programas de saúde, ministrada a docentes de enfermagem no Brasil, comparando o conhecimento pré- e pós-intervenção.

**Métodos:**

estudo de viabilidade realizado em duas universidades brasileiras. Dezesseis professores foram recrutados e participaram de uma intervenção multicomponente que abordou o conteúdo em duas fases: dez sessões pré-gravadas e leituras; cinco sessões interativas online ao vivo. Os dados de conhecimento e aceitabilidade foram analisados descritivamente, e análises de teste t também foram calculadas para determinar a diferença nas pontuações de conhecimento e habilidade.

**Resultados:**

os resultados indicam que os participantes consideraram a intervenção educacional aceitável e viável. No entanto, também expressaram preocupação com o tempo insuficiente para se dedicarem totalmente à intervenção devido às responsabilidades profissionais, enfatizando a necessidade de uma sessão adicional de perguntas e respostas para reforçar o suporte à aplicação prática do conteúdo.

**Conclusões:**

nossos resultados sugerem que a intervenção é aceitável e viável, e apoiarão a expansão dos testes de avaliação por meio de um ensaio clínico em larga escala.

## RESUMEN

**Objetivos:**

determinar la aceptabilidad y viabilidad de una intervención educativa de planificación de programas relacionados con la salud impartida a profesores de enfermería en Brasil, comparando el conocimiento pre y post aprendizaje.

**Métodos:**

estudio de viabilidad realizado en dos universidades brasileñas. Se reclutaron dieciséis profesores, quienes se sometieron a una intervención multicomponente que abordó el contenido en dos fases: diez sesiones pregrabadas y lecturas; y cinco sesiones interactivas en línea en vivo. Los datos de conocimiento y aceptabilidad se analizaron descriptivamente, y también se calcularon pruebas t para determinar la diferencia en las puntuaciones de conocimiento y capacidad.

**Resultados:**

los hallazgos indican que los participantes consideraron la intervención educativa aceptable y factible. Sin embargo, también expresaron preocupación por la falta de tiempo para involucrarse plenamente en la intervención debido a sus responsabilidades laborales, y enfatizaron la necesidad de una sesión adicional de preguntas y respuestas para reforzar la aplicación práctica del contenido.

**Conclusiones:**

nuestros hallazgos sugieren que la intervención es aceptable y factible, y respaldarán la expansión de las pruebas de evaluación a través de un ensayo clínico a gran escala.

## INTRODUCTION

Nursing research plays a central role in providing the scientific foundation for practice. It employs a diverse range of philosophical and theory-based approaches, along with various methodologies, to investigate numerous aspects of healthcare. Thus, it is generally expected that healthcare providers not only engage in evidence-informed practice but also undertake research, based on scientific and aesthetic knowledge^(1)^. When nurses engage in research, production, and evidence-based practice, they contribute to improved quality of patient care, greater efficiency in clinical task performance, and a reduction in overall healthcare costs and expenditures^(2-5)^.

Active and consistent engagement in the research process enhances nurses’ confidence in delivering patient care, increases job satisfaction, improves their ability to read and interpret research evidence, and fosters a more positive attitude toward evidence-based practice^(6)^. However, despite the well-documented benefits of nurses’ engagement in research, those working in lowand middle-income countries often participate less in clinical research projects compared to their medical co-workers^(6)^.

Across Canada and other high-income countries, nursing faculty strive to support not only students, but also colleagues in acquiring these research skills. Within the classroom, this is achieved through progressive course assignments, group work, didactic teaching, and other interactive activities for students to develop knowledge related to research methods, which can then be applied to study design^(7)^. Outside of the classroom, faculty consistently provide mentorship to co-workers in the area of research methodology and design, which promotes the development of skills related to: communication; attention to detail; critical thinking; statistical and graphical analysis of data; and organization, planning, and scheduling^(7)^.

This approach to supporting, training, and mentoring in the area of nursing research does not appear to be consistently provided globally. For instance, in Brazil, among nursing faculty, there is a constant search for strengthening research skills. However, due to a lack of funding and inadequate research infrastructure, there remains a persistent lack of training within this area^(8)^. Moreover, a deficit in research mentorship and training among Brazilian nursing faculty has been shown to be a result of not fully understanding the process, as well as a lack of time, inadequate resources, and underdeveloped research infrastructure^(9)^.

To address these challenges, it is essential to implement targeted educational interventions that equip nursing faculty with practical tools and structured approaches to health-related program planning. Such training fosters a deeper understanding of how to design, implement, and assess health interventions that are contextually relevant and evidence-based. Besides that, enhancing nursing faculty’s research capacity is fundamental for advancing the discipline and improving healthcare outcomes through knowledge translation of results into clinical practice^(10)^.

Research capacity building is particularly important in settings where faculty are expected to lead or collaborate on research and program development initiatives but lack prior exposure to formal training in health planning frameworks and methodologies^(11)^. When educators are actively involved in the development of applied research projects, they not only strengthen their own academic practice but also serve as key facilitators in bridging the gap between research and clinical care^(11,12)^.

Therefore, assessing the acceptability and feasibility of educational interventions among nursing faculty in Brazil can generate critical insights into how best to support research capacity building in low-resource academic environments. Acceptability reflects the perceived relevance, clarity, and usefulness of the content delivered, whereas feasibility addresses the practicality of implementing the intervention given existing institutional constraints^(13)^. By focusing on these dimensions, researchers may identify, in collaboration with clinical nurses, key facilitators and barriers to integrating research and planning competencies within nursing education, and contribute to scaling up strategies for evidence-based practice and knowledge translation^(14)^.

Furthermore, it is highly recommended to determine the acceptability and feasibility of an intervention before larger-scale effectiveness testing can be conducted, avoiding waste^(15)^.

## OBJECTIVES

To determine the acceptability and feasibility of a program planning education intervention delivered to nursing faculty working at two Brazilian universities, comparing participants’ preand post-learning knowledge.

## METHODS

### Ethical considerations

This study received research ethics approval from all three sites involved. The Brazilian and Canadian ethics guidelines in the conduct of the research were adhered to throughout all aspects of this study.

### Study design, period, and location

A feasibility study was used to address the study purpose at two Brazilian universities from September to November 2024. This study was outlined according to the CONsolidated Standards Of Reporting Trials - extension to randomised pilot and feasibility trials^(16)^. Thus, the acceptability of the program planning education intervention and the feasibility of the design need to be assessed before the intervention can be implemented on a larger scale.

### Population or sample, inclusion and exclusion criteria, recruitment

The sample included nursing faculty from two publicly funded Brazilian universities, which together employed 78 nursing professors. Eligible participants were permanent or collaborating professors in a Graduate Nursing Program. Professors on any type of leave during the intervention period were excluded. Data on participant characteristics were collected and analyzed to assess how well the sample represented the target population.

This study employed purposive sampling, which focused on recruiting participants who met the stud’s inclusion and exclusion criteria^(17)^. Recruitment involved email invitations to nursing faculty members who were currently teaching, social media posts, and snowball sampling in which participants referred other potentially eligible individuals to the study^(18)^. All professors from both nursing schools who met the inclusion criteria were invited to participate.

### Study intervention

An online multi-component program planning education intervention was provided to all study participants. The intervention’s goal was to promote knowledge and skills to plan, develop, and assess nursing interventions in randomized controlled trials, pragmatic research, and translational scenarios, according to the Sidani and Braden theoretical framework^(13)^.

The educational content addressed intervention mapping, logic model, implementation and process assessment, interventionist’s role, outcomes, feasibility, and data analysis. Chart 1 reports the details. The intervention was delivered in two phases. Phase one consisted of ten pre-recorded sessions (September and October 2024) and reading of study material, while phase two consisted of five live interactive online sessions (from October 31 to November 6, 2024 - working days only). All participants received a curated list of readings designed to enhance their understanding of the topic.

**Chart 1:** Detailed program content, Rio de Janeiro, Rio de Janeiro, and Campinas, São Paulo, Brazil, 2024

Unit	Content	Implementation
I - Design, implementation, and assessment of interventions or programs	Nurses’ role in the design, implementation, and assessment of interventions or programs (e.g., patient support groups, staff education, new models of care delivery); introduction to assessment; distinction between research and assessment; assessment process overview; importance of understanding the problem requiring intervention; there are three general approaches to understanding the problem that requires intervention: (1) experiential, (2) empirical, (3) theoretical; stating the problem: nature, manifestations, severity level, determinants or causal factors.	Pre-recorded sessions 1 and 2: four hours Live session 1: two hours
II - Intervention mapping and logic model	Mapping for designing or selecting interventions; sources of information on potential interventions; intervention description; integrating information about the problem and the intervention into a logic model; logic model components; logic model use.	Pre-recorded sessions 3 and 4: four hours Live session 2: two hours
III - Intervention implementation	Implementation overview; implementation stages; and resources required for implementation.	Pre-recorded sessions 5 and 6: four hours Live session 3: two hours
IV - Intervention assessment	Aspects or components to be assessed; process assessment methods: document review, observation, self-report; quantitative or qualitative approaches.	Pre-recorded sessions 7 and 8: four hours Live session 4: two hours
V - Outcomes	Outcome definition; types of outcomes (shortand long-term; intended and unintended); evidence required to demonstrate the success of the intervention or program; types of outcome assessment (efficacy and effectiveness); bias or threats to validity; experimental or randomized controlled trials; quasi-experimental or cohort designs; other designs; the role of qualitative methods in outcome assessment; design selection.	Pre-recorded sessions 9 and 10: four hours Live session 5: two hours

Information related to study participants’ age, sex assigned at birth, place of employment, academic title, highest level of education, academic level of students they teach, number of years worked at university, and research focus was collected following recruitment and consent.

We created a virtual classroom to make all the materials available to participants and to allow communication with the main researchers through Google^®^ tools.

### Outcomes

The study measured the acceptability and feasibility of the program planning education intervention, along with professors’ knowledge of the nursing intervention development process. Participants completed demographic and knowledge questionnaires in Portuguese, in addition to their native language. However, the acceptability questionnaire was given in English, as translating it risked reducing its cultural relevance, scientific rigor, and validity.

Intervention acceptability, which corresponds to the degree to which participants agree to participate in, complete, and adhere to the intervention^(13)^, was assessed using a quantitative approach. Researchers used a five-point Likert scale (0=not effective, 1=somewhat effective, 2=effective, 3=very effective, 4=extremely effective). The tool was originally developed and validated in English, with a reported validity score of 0.8^(19)^. Currently, there is no Portuguese version available. However, all study participants were fluent in both English and Portuguese. Higher scores indicate a greater perceived suitability and acceptability of the program planning educational intervention and a higher likelihood of participant adherence. The total score was calculated as the mean of the four-item responses. Participants completed the questionnaire five days after receiving the intervention.

Feasibility, defined as the practicality of implementing the intervention^(13)^, was assessed using both quantitative and qualitative approaches. The quantitative approach was assessed through field diary records, which documented the following information: (1) the number of invited participants and who showed interest in participating; (2) the number of eligible and ineligible professors, and the reasons for ineligibility; and (3) the number of eligible participants who dropped out and the reasons. For the qualitative approach, a debriefing session was held at the end of the final live online interactive session, guided by the open-ended question “What were your impressions of the program planning education intervention?”.

**Figure 1:**
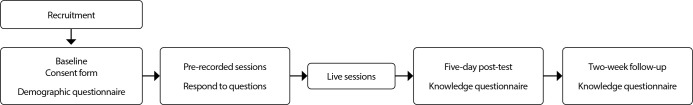
Data collection procedure, Rio de Janeiro, Rio de Janeiro, and Campinas, São Paulo, Brazil, 2024

In terms of knowledge, participants completed an online true-or-false quiz with 15 questions to assess their understanding of the health-related program planning education intervention. True/false questions were created for this study based on the intervention content. They took the quiz three times: before the intervention, five days after, and two weeks after (Figure 1). The questions focused on specific content covered during the intervention. Quiz scores ranged from zero to fifteen.

All data collection tools were uploaded onto a Google Form^®^. Google is the main interface that was used by all research centers of this study. Thus, the three principal investigators have shared access to the data. Across the three sites, the system is secure and involves a two-factor authentication to access the data.

### Implementation

The Canadian researcher, who possesses extensive experience in designing and assessing nursing interventions across diverse settings, conducted the pre-recorded sessions. The Brazilian researchers, native Portuguese speakers with expertise in developing and assessing nursing interventions focused on behavior change among individuals with cardiovascular diseases, led the live sessions and the direct communication with participants. Each live session began with an opening question designed to engage participants and revisit the content covered in the corresponding pre-recorded lecture.

Afterward, participants were divided into small groups to develop a research protocol. Each live session focused on advancing a specific section of the protocol, aligned with the day’s topic. Participants had the autonomy to choose a subject based on shared areas of interest. The researchers guided the process by facilitating comprehension and promoting interaction within each working group.

The entire implementation process occurred online, using the Google Meet^®^ tool.

### Analysis of results and statistics

Acceptability and knowledge data were analyzed descriptively in which measures of central tendency, standard deviation, and frequency values were calculated. One-sample t-test analyses were also calculated to determine the difference in knowledge and acceptability scores according to professional characteristics. We performed a Levene’s test for equality of variances to check the t-test assumptions.

We used Cohen’s d as a measure of effect size following the one-sample t-tests. While a t-test assesses whether the sample mean significantly differs from a specified reference value, Cohen’s d quantifies the size of that difference in standardized units. This provides insight into the practical significance of the findings beyond statistical significance. The Statistical Package for the Social Sciences version 26 was used for data analysis.

Feasibility was assessed using quantitative and qualitative approaches. For quantitative analysis, we measured, in the context of the number of eligible participants, the proportion between the number of professors invited and those demonstrating interest in participating, attrition rates, and contextual effects that interfered with its implementation. A qualitative analysis of the data obtained during the debriefing was conducted. A simple content analysis was used to assess the data obtained from the open-ended question asked to participants at the end of the live sessions^(20)^. The main categories were based on the central topics of the questions that guided the debriefing.

## RESULTS

### Sociodemographic profile

The sample consisted of women with a mean age of 45.4 years (SD=8.3). On average, they had been working at their respective universities for 6.4 years (SD=8.8). Most held a doctoral degree (n=13, 81.3%) and were primarily employed as assistant professors (n=10, 62.3%), teaching either master’s (n=5, 31.3%) or graduate students (n=5, 31.3%). Public health emerged as the most common research focus (n=6, 37.5%) (Table 1).

**Table 1:** Demographic characteristics of professors at both Brazilian universities, Rio de Janeiro, Rio de Janeiro, and Campinas, São Paulo, Brazil, 2024

Variables	n (%)	Mean (SD)
Age		45.43 (8.31)
Sex (female)	16 (100)	
University 1	8 (50)	
University 2	8 (50)	
Assistant professor	10 (62.5)	
Associate professor	4 (25.0)	
Full professor	1 (6.3)	
Doctoral degree	16 (100)	
Post-doctoral degree	3 (18.8)	
Teaching in an undergraduate course	3 (18.8)	
Teaching in master’s or doctoral levels	13 (81.2)	
Time in the university		6.44 (8.81)
Research focus Hospital care Public health Education Worker’s health	10 (68.8) 4 (25.0) 1 (6.3) 1 (6.3)	

### Intervention acceptability

Acceptability score was measured five days after the intervention. They averaged 3.28 (SD=1.31), suggesting participants found the intervention to be very suitable and acceptable.

No significant difference in acceptability scores was noted between the following groups: a. tenured and untenured faculty; b. those who teach at the graduate level and those who teach at the undergraduate level; c. individuals whose program of research addresses public health and those whose program of research address clinical topics; and d. faculty with five years or less of teaching experience and faculty with more than five years of teaching experience at baseline, five days after delivery of the intervention, and two weeks post-intervention.

### Intervention feasibility

**Figure 2:**
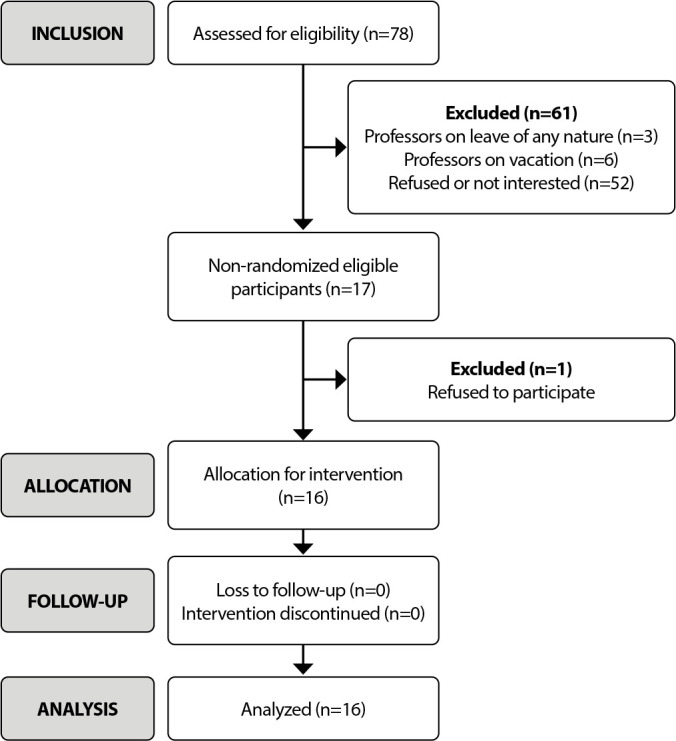
Adapted flowchart based on Consolidated Standards of Reporting Trials - extension to randomized pilot and feasibility trials recommendations^(11)^, Rio de Janeiro, Rio de Janeiro, and Campinas, São Paulo, Brazil, 2024

All professors of both Schools of Nursing were invited to participate. A total of 20 professors expressed interest in participating in the study. However, three of the professors were ineligible (15%) because they were on leave. Of the 17 eligible professors, one (5.8%) refused to participate in the study. Of the 16 professors who were ultimately enrolled in the study, all completed the follow-up, reflecting a zero attrition rate (Figure 2).

A total of 16 professors took part in the debriefing. Analysis of responses to the open-ended question revealed three main topics: limited availability to fully engage in the intervention due to professional obligations; a desire for an additional question and answer session to enhance interaction with the material; and general satisfaction and appreciation for the content delivered.

### Knowledge assessment

Overall, knowledge scores increased from baseline (mean=9.75, SD=1.52) to day five (mean=10.8, SD=1.68). However, a decline was noted two weeks after the intervention, with scores dropping to a mean of 8.62 (SD=1.7).

Moreover, no significant differences in knowledge scores were observed between the following groups: (a) tenured and untenured professor; (b) those teaching at the graduate versus undergraduate levels; and (c) individuals whose research focuses on public health compared to those focused on clinical topics-at baseline, five days post-intervention, and two weeks later (all p > 0.05). Similarly, no differences were found between professors with five years or less of teaching experience and those with more than five years at baseline or the five-day mark (both p > 0.05).

However, at the two-week follow-up, a statistically significant difference emerged between these two experience groups (p=0.014) (Table 2).

**Table 2:** Comparative knowledge with professional characteristics (N=16), Rio de Janeiro, Rio de Janeiro, and Campinas, São Paulo, Brazil, 2024

	Baseline	Five-day follow-up	*p* value^*^	Cohen’s d
	**Mean (SD)**	**Mean (SD)**	**Baseline** **Five-day**	**Baseline** **Five-day**
Knowledge	9.75 (1.52)	10.81 (1.68)		
Academic title Tenure Untenured	10.40 (1.67) 9.45 (1.43)	10.80 (2.16) 10.81 (1.53)	0.886 0.630	1.51 1.74
Teaching Undergraduate course Graduate course	10.0 (1.0) 9.69 (1.65)	11.0 (2.64) 10.76 (1.53)	0.206 0.187	1.57 1.73
Years teaching Five or less More than five	10.10 (1.59) 9.16 (1.32)	10.70 (2.11) 11.00 (0.63)	0.364 0.014	1.50 1.73
Public health^**^ Hospital area^***^	9.83 (1.83) 9.70 (1.41)	10.33 (2.06) 11.10 (1.44)	0.502 0.414	1.57 1.69
University 1 University 2	9.87 (1.35) 9.62 (1.76)	10.50 (1.60) 11.12 (1.80)	0.781 0.912	1.57 1.70

* t-test for equality of variances;

** public health, workers’ health, education;

*** child health, women’s health, oncology, infection prevention and control.

## DISCUSSION

In this study, we presented important findings about a health-related program planning education intervention with evidence for acceptability and feasibility when applied to nursing professors. We observed a significant increase in knowledge scores immediately after the intervention was implemented, despite the small number of participants.

The health-related program planning education intervention was found to be acceptable to all study participants, with a mean score of 3.28 on a 5-point Likert scale. This suggests that the intervention’s content and methods were appropriate and perceived as agreeable and/or satisfactory by the nursing faculty. Therefore, it is quite likely that nursing faculty will apply and integrate the intervention’s content into their curriculum design^(21)^.

This result suggests that the intervention’s content, structure, and delivery aligned with participants’ expectations and professional needs. A mean above the midpoint reflects that most participants perceived the program as appropriate, relevant, and satisfactory. Furthermore, this level of acceptability supports the feasibility of implementing the intervention in similar educational settings and highlights its potential to engage faculty in capacity-building efforts related to planning, designing, and assessing nursing interventions in clinical practice.

Although the intervention content was deemed acceptable, the manner in which it was delivered could be revised to make it more engaging and encourage increased faculty participation. Studies of interventions conducted in different contexts among professors show that institutional culture and whether the intervention is online or in-person may interfere with acceptability^(22,23)^. The inclusion of game-based learning strategies into the live question and answer sessions can serve to not only increase participation but also retention of information leading to increased likelihood for the program planning content to be integrated into curriculum design and/or reviews^(24)^.

In addition, methodological information related to feasibility, like recruitment, retention, data collection, and intervention delivery was also identified. In particular, the anticipated sample size was 28 study participants. However, only 16 nursing faculty (eight from each site) were recruited. This was due to the time with which recruitment occurred. Study participants were recruited over the course of September. This month typically marks the period leading up to midterm exams and the submission of student papers. There are also nursing events at both universities during this period. Thus, faculty workloads are very heavy during this time, leaving little time for research. For a large randomized clinical trial, a plan is needed to recruit participants over a longer period of time, possibly when workloads are lighter (e.g., February to June).

Spreading out recruitment timeline will allow for implementing a more strategic approach to recruiting faculty, such as scheduling recruitment during times when student papers are not due to be submitted or when there are no exams. This can lead to a more diverse and larger sample^(25)^. Thus, the timeline for recruitment will mirror the timeline for an academic semester (five months). It will also allow for two rounds of recruitment, i.e., in the first and second semesters.

In addition to adjusting the timeline for recruitment, ensuring that the time commitment for participant engagement is as short as possible while guaranteeing that data collection is not burdensome will enhance the recruitment and retention of study participants. In this study, only one participant dropped out. This low dropout rate may have been largely due to the ease of completing the data collection tools and the minimal time commitment required^(26)^. Similar data collection methods and tools will be used for randomized control trials. We refined the implementation protocol by assessing these elements in advance, thereby increasing the likelihood of obtaining valid, reliable, and generalizable outcomes. Furthermore, feasibility assessments protect participants and optimize the use of research funding by reducing the risk of failure in later trial phases^(27)^.

Furthermore, knowledge scores increased immediately after the delivery of the intervention and reduced within two weeks following receipt of treatment. This finding aligns with theoretical models of knowledge, which propose that educational interventions lead to immediate changes in knowledge^(28)^. This change in knowledge is sustained over a few days and then decreases over time. For knowledge to remain sustained over longer periods of time, the continued application of that knowledge is required^(28)^. For this study, the application of knowledge occurred during the live sessions. An additional application session will be added to a revised version of the intervention that will be assessed in a larger randomized control trial.

Faculty members with less than five years of teaching experience demonstrated a significantly larger knowledge gain following the intervention. Research on the expertise reversal effect explains this result: novice or lower-knowledge learners benefit more from well-guided instruction, whereas experienced educators may derive less value from highly structured content^(29)^.

In terms of data collection, not all participants completed the questionnaires or actively engaged in the live group discussions. As previously stated, this may have been due to existing work schedules. Additionally, some participants may have been intimidated by the educational content being presented, which addressed program design, implementation, and assessment within the clinical setting. A large portion of the educational intervention focused on research methods of which many study participants may not have had a wholesome understanding and/or may have felt embarrassed or ashamed to ask questions. Thus, there is a need to promote participation in the online group discussions in which an atmosphere of trust and openness is created^(30)^.

This can be achieved by using clear language to ensure that all participants understand the information; practicing active listening to address concerns; creating a supportive online environment that provides a safe and inclusive space for participants to express concerns, ask questions, and learn; and providing regular feedback to study participants during the online discussions^(31)^. To encourage the completion of all items on the data collection tools, remind study participants of the purpose of the study and the importance of completing the questionnaire.

### Study limitations

This research has limitations that should be considered. We highlighted that data collection occurred during a period when nursing faculties were overloaded due to the end of the semester and the submission of final exams and student work. Additionally, numerous events occurred at both universities during this period. These factors may have directly impacted professors’ interest in and acceptance of participating in the study. The use of an English-language instrument to measure acceptability without prior cross-cultural validation may have introduced measurement bias, especially regarding participants’ understanding and interpretation of items. Nonetheless, we have decided to maintain it as the faculty members routinely engage with academic materials and assessment tools in English, which likely minimized comprehension issues and supported the feasibility of its application in this context. Additionally, the self-reported nature of the data increases the risk of social desirability bias, as participants may have provided responses aligned with perceived expectations.

The small sample size restricts the generalizability of the findings and limits the ability to detect subtle variations in responses. Nevertheless, this study is the first to assess the feasibility of an intervention before its implementation on a large scale. The absence of a control group limits the ability to attribute the observed outcomes specifically to the examined intervention or context. A future study with a larger sample and control group is recommended.

Finally, the qualitative analysis, based on a single open-ended question, offers only a partial and potentially superficial insight into participants’ perspectives, restricting the depth and breadth of thematic exploration.

### Contributions to nursing, health or public policy

This study makes a significant contribution to the field of nursing by demonstrating an educational intervention on program planning lasting five days, in addition to boosting professionals’ autonomy in careful planning, systematic assessment, and meaningful translation of nursing interventions for different health conditions and, consequently, increasing national evidence production and high-quality care provision. In addition to the evidence of acceptability and feasibility of the findings of this study, the frequent satisfactory reports from participants (not included in the scope of this research) stand out. This is a viable strategy that could be made available as part of the protocol in this and other institutional settings. For the health field, the possibility to promote knowledge and train professors and researchers to systematically produce effective interventions that directly impact the Brazilian population’s health is notable.

## CONCLUSIONS

This feasibility study assessed the acceptability of a five-day program planning education intervention delivered to nursing faculty teaching at two public universities in Brazil. The challenges on feasibility of recruitment, retention, data collection, and intervention delivery plan were also discussed. The analysis identified the intervention as acceptable and demonstrated that it enhances professors’ knowledge related to planning, designing, implementing, and assessing nursing interventions related to clinical practice. Based on the results of this study, the overall recruitment plan, the timeline for intervention delivery, how often different components of the intervention are provided, and the medium through which the intervention is delivered will be revised and assessed in a larger randomized control trial.

## Data Availability

The research data are available within the article.
